# Immediate newborn care practices delay thermoregulation and breastfeeding initiation

**DOI:** 10.1111/j.1651-2227.2011.02215.x

**Published:** 2011-08

**Authors:** Howard L Sobel, Maria Asuncion A Silvestre, Jacinto Blas V Mantaring, Yolanda E Oliveros, Soe Nyunt-U

**Affiliations:** 1 WHO Representative's Office in the PhilippinesManila, Philippines; 2 Department of Pediatrics, University of the Philippines, Manila – Philippine General HospitalManila, Philippines; 3 Department of Clinical Epidemiology, University of the PhilippinesManila, Philippines; 4 Department of Health, National Centers for Disease Prevention and ControlManila, Philippines

**Keywords:** Breastfeeding initiation, Essential newborn care, Hypothermia, Immediate newborn care practices, Neonatal sepsis

## Abstract

**Aim:**

A deadly nosocomial outbreak in a Philippine hospital drew nationwide attention to neonatal sepsis. Together with specific infection control measures, interventions that protect newborns against infection-related mortality include drying, skin-to-skin contact, delayed cord clamping, breastfeeding initiation and delayed bathing. This evaluation characterized hospital care in the first hours of life with the intent to drive policy change, strategic planning and hospital reform.

**Methods:**

Trained physicians observed 481 consecutive deliveries in 51 hospitals using a standardized tool to record practices and timing of immediate newborn care procedures.

**Results:**

Drying, weighing, eye care and vitamin K injections were performed in more than 90% of newborns. Only 9.6% were allowed skin-to-skin contact. Interventions were inappropriately sequenced, e.g. immediate cord clamping (median 12 sec), delayed drying (96.5%) and early bathing (90.0%). While 68.2% were put to the breast, they were separated two minutes later. Unnecessary suctioning was performed in 94.9%. Doctors trained in neonatal resuscitation were 2.5 (1.1–5.7) times more likely to unnecessarily suction vigorous newborns. Two per cent died and 5.7% developed sepsis/pneumonia.

**Conclusions:**

This minute-by-minute observational assessment revealed that performance and timing of immediate newborn care interventions are below WHO standards and deprive newborns of basic protections against infection and death.

## Introduction

A deadly neonatal sepsis outbreak in one city hospital in the Philippines garnered national attention (1). The authors, who investigated the outbreak, wanted to understand how immediate newborn care practices may impact on neonatal sepsis rates in hospitals nationwide.

Annually, 82 000 of the 2.4 million live births die before reaching their fifth birthday, making the Philippines one of 42 countries accounting for 90% of all global under-five deaths. About half of these deaths occur in the first 28 days (neonatal mortality rate of 16 per thousand live births) and one-quarter in the first 2 days of life. Birth asphyxia, complications of prematurity and severe infections account for the majority of newborn deaths (2–4). The decline in childhood deaths witnessed in 1993–2003 was minimal because of negligible changes in neonatal death rates. If newborn mortality is not reduced more rapidly, the goal of reducing childhood mortality by two-thirds (Millennium Development Goal no. 4) by 2015 will not be met (5).

WHO has identified simple interventions that, if applied routinely, mitigate some of the threats newborns face. These early interventions are integral to hospital infection control practices because they reduce the risk of neonatal sepsis (6,7).

First, hypothermia can threaten newborns with delayed foetal-to-newborn circulatory adjustment, acidosis, hyaline membrane disease, coagulation defects, infection and brain haemorrhage (8). Every second of exposure to the outside environment results in heat loss via evaporation, conduction, convection and radiation. Thorough drying, direct skin-to-skin contact immediately upon delivery and covering with a blanket and bonnet (prior to cord clamping) mitigate this threat (9,10). Drying also stimulates breathing. Sustained skin-to-skin contact also initiates colonization of the newborn with maternal flora (as opposed to hospital flora) and facilitates olfactory learning, successful intake of colostrum and sustained breastfeeding (11,12). Bathing not only exposes newborns to hypothermia, but also removes maternal bacteria and the vernix caseosa (a potent inhibitor of *Escherichia coli*) (13), and eliminates the crawling reflex (14).

**Key notes**

- Crucial interventions in the immediate period after birth should be properly timed and sequenced to enable early thermoregulation and appropriate breastfeeding initiation.

Second, while judicious use of resuscitation by skilled staff is essential, suctioning can cause apnoea, vagal-induced bradycardia, slower rise in oxygen saturation (15), and possible mucosal trauma with increased risk of infection. Routine suctioning of the newborn should be stopped (16,17).

Third, delaying cord clamping until cord pulsations stop, typically around one to three minutes, reduces the risk of anaemia (18,19). Furthermore, in preterm infants, delayed cord clamping is associated with fewer transfusions and fewer intraventricular haemorrhages (20).

Fourth, initiation of breastfeeding within the first hour reduces the risk of infection-related death and increases the likelihood of sustained breastfeeding (21,22). Babies typically are ready to initiate breastfeeding between 20 and 60 min (14).

Finally, weighing, examining and providing vitamin K injections and hepatitis B vaccinations, while essential, should not interfere with the early, time-sensitive actions (23). Evidence further shows that offering these interventions as a package of services together with hospital infection control measures increases effectiveness (24).

Less than half (43%) of all Philippine deliveries occur in hospitals (3). Nevertheless, crowding and understaffing of birthing facilities are common, as in many developing countries. The consequent congestion may contribute to suboptimal newborn care practices and subsequent neonatal sepsis. However, data about newborn care practices are sparse. About half (54%) of newborns initiated breastfeeding in the first hour of life (3). Data on cord clamping, drying and immediate skin-to-skin contact are anecdotal. Furthermore, hospital reports of neonatal deaths and sepsis are incomplete at the national level.

The goal of this evaluation was to characterize minute-by-minute newborn care done in the first hours of life in 51 large hospitals in the Philippines. These data will be used to drive policy change, strategic planning and hospital reform.

## Methods

### Evaluation design

A detailed observational assessment of immediate newborn care practices was performed on consecutive deliveries in each selected hospital during the fourth quarter of 2008. Data on total hospital deliveries, live births, deaths and sepsis/pneumonia were abstracted from annual reports.

### Assessment tool and protocol development

Intrapartum assessment tools were developed through collaboration between the University of the Philippines – Philippine General Hospital, WHO, and the Department of Health (DOH) (Please see http://www.wpro.who.int/NR/rdonlyres/94E9DB1A-1047-4541-BE98-3E8A71773857/0/Delivery_Assessment_Tool_EINC_Philippines.pdf). Physician assessors (paediatricians and internists) were trained to use the tool, secure informed consent, administer the questionnaire and conduct interviews. Pre-testing of the assessment tool was performed in two hospitals in Manila.

### Facility selection

The largest 150 government hospitals were identified and nine (of 17) regions were selected per previous protocol (25). Fifty hospitals were then selected from these regions by random sampling using the Excel RAND function. The single largest delivery centre in the country was added purposively. This was the greatest number of hospitals and regions that could be feasibly studied.

### Subject selection

After informed consent, consecutive births were observed within a 24-h period with the intent to observe 10 mother–baby dyads per hospital. Physician assessors started observations with the first delivery of their shift. For hospitals with fewer than 10 births at the end of the 24-h recruitment period, additional births were included from a neighbouring, higher volume hospital already on the selection list to make up for the shortfall. For practical reasons, at the end of 24 additional hours, recruitment was stopped. For multiple births, only the first newborn was included. Infants with obvious lethal deformities were excluded.

### Data collection

Trained physicians completed the assessment tool to document the minute-by-minute sequence of events and interventions occurring just prior to delivery to the first hours after birth and rooming-in. They were instructed to observe only, without commenting or intervening. Health staff were unaware of the practices being observed. The direct observations recorded the performance and timing of immediate newborn care interventions. Time of skin-to-skin contact, breastfeeding, weighing, examination, vitamin K injection and hepatitis B vaccination, eye prophylaxis and rooming-in were recorded to the minute. Time of drying and cord clamping were recorded to the second. Details of rooming-in and breastfeeding initiation that occurred after the 2-h direct observation period were obtained from records or health staff interviews. A brief questionnaire was administered to the attending health provider to determine his or her training background.

### Data management and analysis

Data were presented as percentages for the categorical variables and median ± interquartile range for times. All variables were analysed by subgroup, including training of the delivery attendant, mode of delivery and hospital size. Sensitivity analysis was carried out by replacing missing data with the largest and smallest value in the data set. Analysis was performed using Stata version 9.1. (Stata Corp, College Station, TX, USA).

### Ethical considerations

Informed consent was secured from each mother prior to the second stage of labour to assure confidentiality and anonymity for the data collected. Women presenting to the hospital during the second stage of labour were excluded. Ethical review was secured from the Institutional Review Board, National Institutes of Health, Manila.

## Results

### Hospital records review (2007)

Of the 201 760 deliveries, 197 328 (97.8%) were live births and 43 373 (21.5%) were delivered by caesarean section. Of the live births, 11 003 (5.7%) infants developed neonatal sepsis/pneumonia and 3980 (2.0%) died. A large variation among each of the indicators existed across the 51 hospitals (Table 1). The smallest hospital, a tertiary perinatology centre, had 479 deliveries and the largest, an exclusive maternity hospital, 25 246. Three hospitals reported no neonatal deaths, and six reported no sepsis or pneumonia cases.

**Table 1:** Variation in deliveries across 51 hospitals

Characteristics	Median	Inter-quartile range
Total annual deliveries, n	3330	2277–5199
Caesarean section, n (%)	794 (22.3%)	292–1364 (15.9–31.4%)
Total live births, n	3385	2217–5003
Neonatal mortality, n (%)	55 (1.8%)	18–99 (0.8–2.9%)

### Observational assessment

A total of 481 mother–baby pairs were observed between 19 October and 28 December 2008. A minimum of five and a maximum of 16 consecutive deliveries were observed per hospital. Of the 481 newborns, 67 (14.1%) were low birth weight (<2.5 kg), 6 (1.3%) very low birth weight (<1.5 kg) and 43 (9.0%) macrosomic (≥3.8 kg).

Obstetricians attended 374 (77.8%) deliveries, midwives attended 62 (13.0%) and non-obstetric physicians 43 (8.9%). Although monitored during the first stage of labour, one subject delivered in an elevator, unattended. Paediatricians attended 209 (43.5%) of newborns, nurses 169 (35.1%), midwives 63 (13.1%), obstetricians 19 (4.0%) and other doctors 15 (3.1%). Delivery was by caesarean section in 107 (22.2%), forceps in 7 (1.5%) and spontaneous vaginal in 367 (76.3%).

### Performance of appropriate interventions

**Figure 1:**
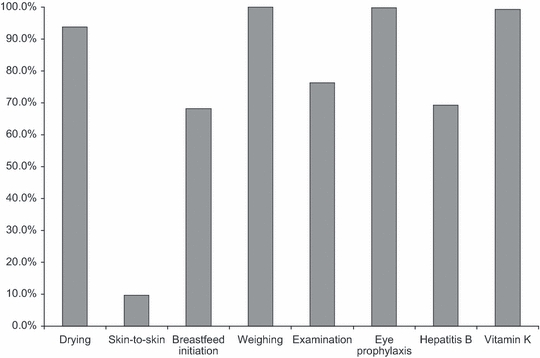
Performance of appropriate newborn care interventions (N = 481).

More than 90% of infants were dried, weighed, given eye prophylaxis and injected with vitamin K. Approximately 70% were put to the breast, examined and vaccinated against hepatitis B virus. Less than 10% were allowed skin-to-skin contact (Fig. 1).

### Performance of inappropriate interventions

**Figure 2:**
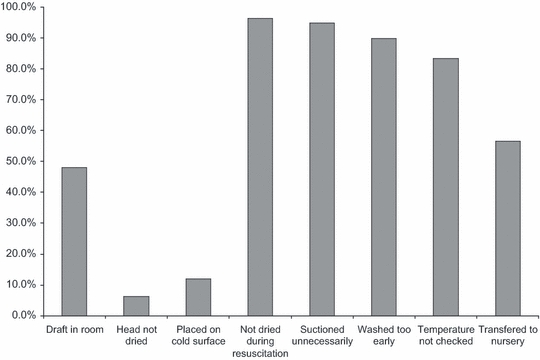
Performance of potentially harmful newborn care practices (N = 481).

Early bathing, non-immediate drying, placement on a cold surface and transfer to a nursery were commonplace (Fig. 2). Of the 455 newborns who were breathing spontaneously at delivery, 432 (94.9%) were suctioned, with 363 (84.0%) suctioned more than once. Of the 26 newborns (5.4%) that had no breaths or occasional gasping, only 1 (3.9%) was dried during the sequence (Fig. 2).

### Sequence of interventions (median time to start of intervention)

**Figure 3:**
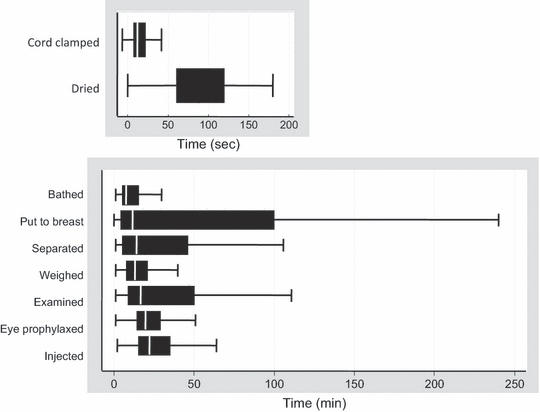
Sequence of interventions conducted within the first hours of life (median, 25%/75% interquartile and max/min adjacent values).

Cords were clamped immediately (12 sec), newborns were then dried (1 min), bathed (8 min), put to the breast (10 min), separated (12 min) for weighing (13 min), physical examination (17 min), eye prophylaxis (20 min), transfer to a ‘nursery’ (20 min), injections (22 min) and then returned to their mothers (2 h and 35 min). A median duration of only 2 min was allowed for their first colostrum feed. Wide interquartile ranges were observed (Fig. 3).

### Attendant training

Nearly half of the attendant paediatric staff was trained in neonatal (236, 49.1%) and paediatric resuscitation (208, 43.2%) while only 75 (15.6%) in infection control and 11 (2.3%) in infant and young child feeding.

### Subgroup analyses

All variables were assessed by previous training, modes of delivery and hospital size. Those trained in neonatal resuscitation were 2.5 (1.1–5.7) times and in paediatric resuscitation 2.2 (0.96–5.2) times more likely to unnecessarily suction babies who were already breathing. Having no attendant training correlated with breastfeeding initiation within one hour or a duration longer than 20 min. Timing of the physical examination was affected by a maximum of 23 min when comparing groups trained or not trained in resuscitation. The timing of other interventions varied by a maximum of 4 min. Comparisons between abdominal and vaginal delivery and between hospitals with more than vs. fewer than 3000 deliveries a year showed a maximum difference of 2 min for all variables except for cord clamping (which did not differ).

### Missing data and sensitivity analysis

One hospital annual report was missing data for number of deliveries and live births, eight were missing data for caesareans, five for neonatal deaths and 14 for sepsis/pneumonia. After replacing all missing data with the minimum and maximum values, the median number of deliveries and live births changed a maximum of 147, the caesarean section rate a maximum of 2.5% and the neonatal mortality rate 0.4%. Sepsis/pneumonia ranged 1.3–11.4% after similar sensitivity analysis.

For the observational assessments, no data were missing for performance of each intervention. One (0.2%) had missing data on delivery attendant and 6 (1.3%) for attendant of the newborn. Missing data on timing of interventions produced a maximum of 10.1%. Median and interquartile ranges were calculated assuming all missing data were either the smallest or the largest value of the variable. In no case did this cause the median to change.

## Discussion

Available 2007 hospital records revealed neonatal sepsis/pneumonia and mortality rates of 5.7% and 2.0%, respectively. Many interventions, such as drying, weighing, examining, providing eye prophylaxis and supplying vitamin K, were observed. Timely birth doses of hepatitis B vaccine tripled from baseline levels (25). Unfortunately, these interventions were performed in sequences that did not allow the newborns to benefit from *all* of their mothers’ natural protection in the first hour of life, i.e. provision of warmth, blood transfusion from the placenta, protection from infection via skin-to-skin contact and completion of colostrum feeding (8,12,18,19,21,27). Cohorting newborns in ‘nurseries’ for mandatory observation periods and clearance exposed them to hospital-acquired flora.

Hospital practices permitted the overwhelming majority of newborns to be exposed to cold, similar to practices in other countries (27). Only one of 26 newborns with apnoea was dried. Most newborns with primary apnoea will start breathing from stimulation during drying.

This evaluation found that 68.2% were put to the breast, similar to national survey results (3). However, this process was not optimal because the infants’ mouths were pried open, positioned onto the areola and their cheeks were stroked to trigger rooting only 10 min after birth, a time before newborns are ready to breastfeed (14). They were allowed only two minutes of this forced attachment.

The Academy of Breastfeeding Medicine (23) states that, for a healthy newborn, procedures should be delayed to allow early parent–newborn interactions and the first breastfeed. Newborns were typically separated for weighing, examination, eye prophylaxis and injections at only 12 min after delivery. The majority (88%) were reunited with their mothers at 155 min, a time when newborns younger than 24 h are usually asleep. When newborns eventually initiate breastfeeding, the risk for infection-related death is doubled or tripled (21).

The need for basic life-saving interventions and for beneficial parent–newborn interactions indicates that procedures carried out immediately after delivery should be standardized in time and order. Unnecessary procedures, such as routine suctioning, early bathing and separation of newborns from their mothers, should be discontinued. Aside from potential for harm, these procedures burden already overworked hospital staff.

These findings should not be surprising. Pre- and in-service training in medical and allied schools do not address key WHO guidelines in newborn care (28). WHO guidelines have not emphasized the importance of the timing of early interventions for newborns. Even with formal training, optimal outcomes may not be realized (29). This is particularly true when the physical and policy environments do not enable appropriate newborn care practices or disable outdated and inappropriate practices.

This evaluation was limited to the largest hospitals in only nine of the 17 regions. However, the deliveries in the 51 hospitals accounted for more than 10% of the deliveries nationwide. It did not include either home deliveries or those in smaller centres. We focused on the large hospitals because changing practices here will affect training of obstetric/paediatric residents and midwifery/nursing/medical students.

Extraction of data from hospital records was limited by problems inherent in hospital recording. Many hospitals had missing data for key outcome variables. The sensitivity analysis revealed that, regardless of missing data, a high burden of disease exists in these large hospitals. We could not validate reports of zero deaths or sepsis. Accurate reporting of sepsis cases was limited by variation in interpretation of clinical presentations and laboratory results. While gestational ageing techniques were not uniformly performed in the hospitals, birth weight categories were available.

Despite the Hawthorne effect implicit to the observational methodology, serious hospital practice issues were evident. The problems are likely to be more severe than our evaluation uncovered. Recording bias is another potential limitation for data on rooming-in and breastfeeding after the 2-h observation window.

A secondary analysis of a nationwide survey revealed death rates of neonates born to women delivering in a healthcare facility to be statistically similar to those born at home attended by a non-health professional (OR 1.0; 95% CI, 0.63–1.57) (26). This comparative data together with the findings of the present evaluation set off a series of responses to address inappropriateness and lack of standardization of immediate newborn care practices. The Department of Health convened a technical working group to review current evidence and draft evidence-based recommendations. The resulting Essential Newborn Care protocol then underwent expert and stakeholder panel review in a guideline development process. It specifically defined the time for each intervention and made explicit statements to stop unnecessary interventions. It has now become an official publication (30). The DOH is spearheading strategies to (i) jumpstart the hospital reform agenda as the next phase of health sector reform; (ii) develop model hospitals and networks of excellence in each of 17 regions; (iii) update the pre-service and in-service medical, nursing and midwifery curricula and (iv) conduct a nationwide social marketing campaign. Preliminary results show that the one hospital that implemented the protocol has seen historically low neonatal deaths.

Globally, 450 newborns die every hour (5). Their limited reserves and defences make newborns vulnerable to attendant practices. The scope and seriousness of threats, even with skilled attendants, were not clear until embarking on this direct observational assessment. We believe this is the first evaluation of its kind that enables quantification of the timing and performance of various interventions.

Although standards for immediate newborn care exist in high- and low-income countries alike, direct observational studies may uncover substandard practices. Even in developed countries, the sequence and timing of critical interventions may still require changes or standardization.
